# Austenite Stability and Deformation Behavior in Medium Mn Steel Processed by Cyclic Quenching ART Heat Treatment

**DOI:** 10.3390/ma14237132

**Published:** 2021-11-23

**Authors:** Chunquan Liu, Fen Xiong, Guanni Liu, Yong Wang, Yuxin Cao, Zhengliang Xue, Qichun Peng

**Affiliations:** 1 Hunan Institute of Technology, Hengyang 421002, China; xiongfen93@163.com (F.X.); lgnaishishi1128@163.com (G.L.); wangyong0911@wust.edu.cn (Y.W.); caoyuxin@wust.edu.cn (Y.C.); 2 The State Key Laboratory of Refractories and Metallurgy, Key Laboratory for Ferrous Metallurgy and Resources Utilization of Ministry of Education, Wuhan University of Science and Technology, Wuhan 430081, China; pengqichun1964@163.com

**Keywords:** medium Mn steel, retained austenite, discontinuous TRIP effect, tensile properties, work hardening

## Abstract

This study investigated the austenite stability and deformation behavior of cyclic quenching-austenite reverse transformation processed Fe-0.25C-3.98Mn-1.22Al-0.20Si-0.19Mo-0.03Nb medium Mn steel. A number of findings were obtained. Most importantly, the extent of the TRIP effect was mainly determined by an appropriately retained austenite stability rather than its content. Simultaneously, chemical elements were the key factors affecting austenite stability, of which Mn had the greatest impact, while the difference of retained austenite grain size and Mn content resulted in different degrees of retained austenite stability. Additionally, there were still large amounts of strip and granular-retained austenite shown in the microstructure of the CQ3-ART sample after tensile fracture, revealing that the excessively stable, retained austenite inhibited the generation of an extensive TRIP effect.

## 1. Introduction

Lightweight automobiles are an effective means to reduce energy consumption and environmental pollution [1,2,3]. Advanced high-strength steel, especially advanced high-strength medium manganese steel for third-generation automobiles, is currently the most promising lightweight automotive material [4,5]. Research on the strength-ductility mechanism of automotive steel has shown that it effectively and simultaneously improves the strength and plasticity of automotive steel by using phase-transformation-induced plasticity [6,7,8]. Metastable austenite plays a vital role in improving the properties of steel. Metastable retained austenite deforms under the action of an external load, and then induces the formation of martensitic transformation, thereby improving the material strength. Simultaneously, the volume of the martensite phase is larger than that of the austenite phase; thus, the continuously generated martensite increases in volume and compresses the surrounding phases (such as ferrite/austenite), causing successive plastic deformations in the surrounding phases [9,10]. In addition, the strain-induced transformation from austenite to martensite produces stress relaxation, which delays the occurrence of material necking and increases the strength and plasticity of the material [11,12].

Lee et al. [13] found that the coarse austenite growing on the Mn-rich segregation band of hot-rolled Fe-0.1C-10Mn-1Si-0.3Mo-0.5V (wt%) medium manganese steel can more effectively stimulate phase transformation and induce plasticity. Moreover, the Mn content in austenite has a greater influence on its stability than austenite grain size, which leads to an extensive TRIP effect in the austenite of Mn-rich strips, thus obtaining ultra-high strength automobile cold-rolled steel plates. Cai et al. [14] studied the stability and deformation behavior of austenite in Fe-11Mn-4Al-0.2C (wt%) medium Mn steel, and the authors considered that grain size is the key factor controlling the stability of austenite, while the optimum grain size for the highest austenite stability is 0.6 μm. Li et al. [15] found that the austenite stability in Fe-0.18C-11Mn-3.8Al (wt%) medium Mn steel is mainly controlled by austenite grain size rather than austenite grain orientation, and that the excellent elongation of medium Mn steel is related to the high austenite stability and the synergistic deformation of ferrite.

The purpose of this study was to lay a scientific foundation for obtaining excellent comprehensive performance in Nb-Mo medium Mn steel by the cyclic quenching-austenite reverse transformation (CQ-ART) process on the basis of our previous research [16]. The focus of the study was improving retained austenite content and stability after the CQ-ART process. In addition, the microstructure evolution, tensile properties, and work-hardening behavior of Nb-Mo medium Mn steel were described the influence of the microstructure on retained austenite was explored; and the deformation mechanism of the cold-rolled experimental steel was analyzed to further explore and explain the mechanism of the TRIP effect.

## 2. Experimental Procedure

**Figure 1:**
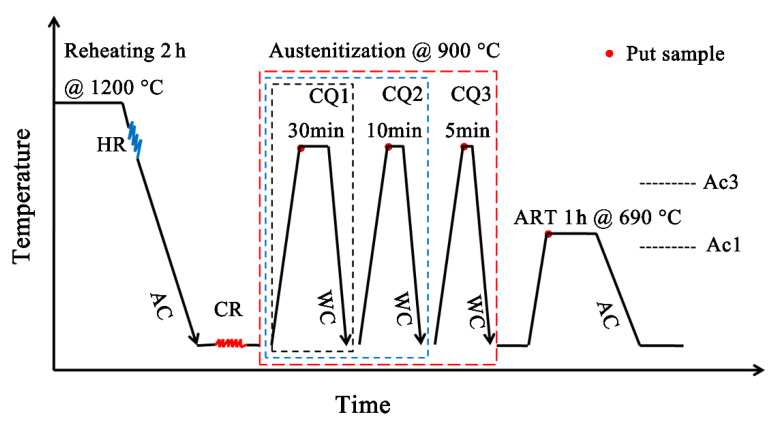
CQ-ART heat treatment process schematic diagram of cold-rolling experimental steel.

The investigated alloy with an actual chemical composition of Fe-0.25C-3.98Mn-1.22Al-0.20Si-0.19Mo-0.03Nb (wt%) is shown in Table 1, and it was melted using an intermediate frequency induction furnace. The AC_1_ and AC_3_ temperatures of the experimental steel were measured in our previous work [16]; at the same time, the highest retained austenite content was obtained at the intercritical annealing temperature of 690 °C. The bullet-shaped billet was homogenized at 1200 °C for 2 h, hot-forged into a rod of section size of 30 mm (wide) × 30 mm (thick), and then air-cooled to ambient temperature. Subsequently, the rods were cold-rolled to 3.8 mm thick strips, air-cooled to ambient temperature, and then the as-hot-rolled strips were cold-rolled to 1.9 mm in thickness. The schematic diagram of the CQ-ART heat treatment process of the experimental steel is shown in Figure 1. Three processes of cold-rolling experimental steels were separately carried out as follows: CQ1 experimental steel was rapidly reheated to 900 °C for 30 min, followed by being water-quenched to ambient temperature; on the basis of process CQ1, the experimental steel was rapidly reheated to 900 °C for 10 min, and then water quenched to ambient temperature (CQ2); on the basis of process CQ2, the experimental steel was rapidly reheated to 900 °C for 5 min, and then water-quenched to ambient temperature (CQ3); finally, the three experimental steels treated with different processes were intercritical annealed at 690 °C for 1 h, and then air-cooled to ambient temperature.

The microstructure was characterized by field-emission scanning electron microscopy (SEM; FEI, Hillsboro, OR, USA), field-emission transmission electron microscopy (TEM; JEOL, Tokyo, Japan), and X-ray diffraction (XRD; Bruker, Karlsruhe, Germany). The chemical compositions in austenite and ferrite phases were determined by energy-dispersive spectroscopy (EDS) in the TEM. Austenite content was determined by X-ray diffraction (XRD) based on the integrated intensities of (200)*α*, (211)*α*, (200)*α*, (220)*γ*, and (311)*γ* diffraction peaks [17,18], and was calculated using Equation (1).

(1) $$V_{\gamma }=1.4I_{\gamma }/(I_{\alpha }+1.4I_{\gamma })$$

where *I_γ_* is the integrated intensity of austenite and *I_α_* is the integrated intensity of ferrite.

In addition, austenite grain size was measured by TEM-Digital Micrograph software (DM; Ametek, Middleboro, MA, USA).

## 3. Results and Discussion

### 3.1. Microstructure Evolution and Mechanical Properties

**Figure 2:**
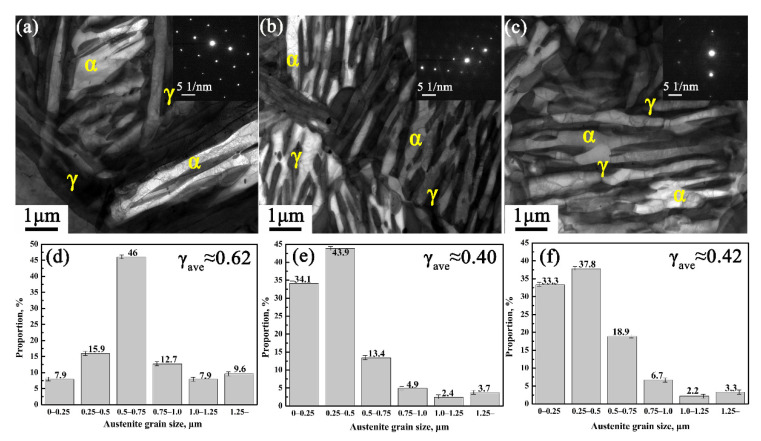
TEM microstructure and grain size of experimental steel with different numbers of quenching cycles: (**a**,**d**) CQ1-ART, (**b**,**e**) CQ2-ART, and (**c**,**f**) CQ3-ART.

Figure 2 shows the TEM microstructure and austenite grain size of the three CQ-ART samples. It can be seen from Figure 2a–c that the microstructure of the three CQ-ART samples mainly consisted of retained austenite and ferrite. Thirty TEM images were used to characterize austenite grain size by Digital Micrograph software (DM; Ametek, Middleboro, MA, USA). Retained austenite grain size decreased with the increase in the amount of cyclic quenching, i.e., it decreased from 0.62 μm for the CQ1-ART sample to 0.42 μm for the CQ3-ART sample (see Figure 2d–f). It was preliminarily speculated that austenite grains could be refined by increasing the amount of cyclic quenching. For the CQ3-ART sample, the grain size of its microstructure was smaller than that of the CQ1-ART sample, obviously, but equivalent to the CQ2-ART sample, which indicated that the trend in grain size change in experimental steel stabilized after three quenching cycles.

**Figure 3:**
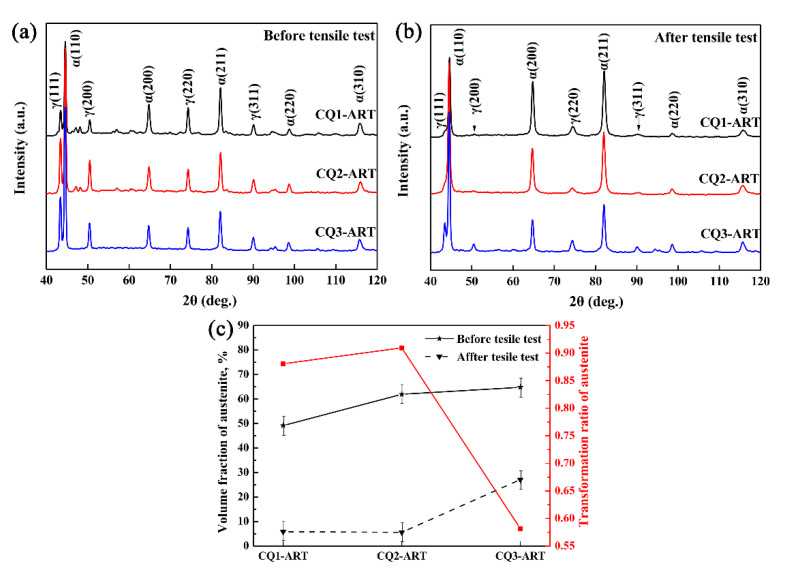
XRD characterization of experimental steel: (**a**,**b**) XRD patterns and (**c**) measured austenite fractions and transformation ratio of austenite.

Figure 3a–c shows the XRD patterns and measured austenite fractions of the three CQ-ART samples. Before the tensile tests, the calculated values of retained austenite in the CQ1-ART, CQ2-ART, and CQ3-ART specimens were 49.2%, 62.0%, and 64.8%, respectively; the calculated retained austenite values after the tensile tests were 5.9%, 5.6%, and 27.1%, respectively; and the calculated transformation rates of retained austenite were 88%, 90%, and 58%, respectively. With the increasing number of quenching cycles, the retained austenite content increased, and the retained austenite content in the CQ3-ART sample reached the highest value (64.8%). It is considered that the multiple quenching cycles effectively reduced the austenite grain size, thereby increasing the C and Mn contents in the austenite, improving the retained austenite stability, and ultimately increasing the retained austenite content at room temperature. Thus, reducing the retained austenite grain size is a feasible way to improve its stability.

**Figure 4:**
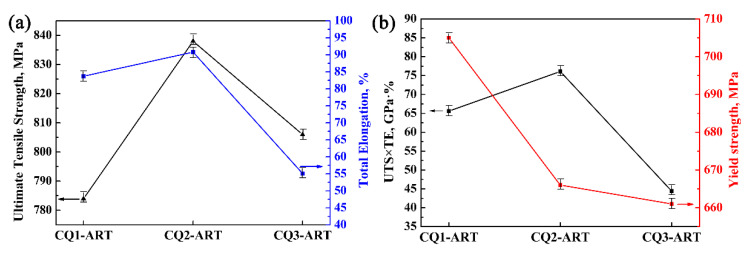
Tensile properties of experimental steel with different numbers of quenching cycles: (**a**) UTS and TE; (**b**) UTS × TE and YS (UTS: ultimate tensile strength, TE: total elongation, YS: yield strength).

Figure 4 shows the tensile properties of the three samples in the CQ-ART process. With increasing the number of quenching cycles, the ultimate tensile strength, total elongation and product of strength, and elongation of the experimental steel initially increased, and then decreased. The tensile strengths of the three samples were similar, while the elongation of the CQ3-ART sample was reduced by 35.7% compared with that of the CQ2-ART sample. Combining Figure 3c and Figure 4, it can be seen that the austenite transformation rate of the CQ3-ART sample with the highest retained austenite content was only 58%, while 27.1% remained untransformed. These results reveal that the mechanical properties of the experimental steel not only related to the retained austenite content, but also to the retained austenite transformation rate. It is considered that the elongation is indirectly determined by the amount of retained austenite transformation. The CQ2-ART sample exhibited the best mechanical properties, i.e., an ultimate tensile strength of 838 MPa, a total elongation of 90.8%, and a product of strength and elongation of 76.1 GPa·%, which meet the performance requirements of advanced high-strength steel for the third generation of automobiles (>30 GPa%). In summary, the CQ2-ART sample was accompanied by a high retained austenite content and the highest retained austenite transformation rate, thus producing a more extensive TRIP effect, thereby improving the strength-plasticity of the experimental steel.

### 3.2. Austenite Stability

From different perspectives, austenite stability has different understandings: 1. From the perspective of macro application of materials, austenite stability refers to the stability of whole austenite in the materials; thus, the evaluation standard should be an average performance of all austenite stabilities in the structure. 2. From the perspective of the micro scale of materials, austenite presents a different morphological distribution, such as block or film, and different forms of austenite exhibit different transformations during deformation, showing different stabilities. In the design of advanced high-strength steel for the third generation of automobiles, it is particularly important to use the phase transformation of retained austenite to enhance the plasticization and strengthening effects of the material. Simultaneously, the strength and elongation improvement of third-generation automotive advanced high-strength steel is related to retained austenite content and stability. The retained austenite stability includes two parts: thermodynamic stability and mechanical stability. Ms is used to characterize the thermodynamic stability of the retained austenite during martensite transformation during cooling, and a low Ms temperature indicates a high stability of the retained austenite [19,20,21]. $M_{S}^{\sigma }$ is used to evaluate the mechanical stability of the retained austenite during stress-induced martensite transformation, and is the starting temperature of martensite transformation when a fixed stress value is applied [22].

The effect of alloying elements on austenite stability mainly depends on the starting temperature of martensite transformation. In our previous article, we calculated that the average Mn concentrations in the retained austenite of the CQ1-ART, CQ2-ART, and CQ3-ART samples were 6.16, 7.31, and 7.19 wt%, respectively, while the average C concentrations in the retained austenite were 0.5171, 0.5139, and 0.5124 wt%, respectively. The martensite start temperature was estimated by using Equation (2) [23]. According to the calculated C and Mn contents, when the C contents in the austenite were similar, the Mn contents in the three samples had a strong influence on the Ms point temperature.

(2) $$M_{S}=539.423C-30.4\mathrm{Mn}-7.5\mathrm{Si}+30\mathrm{Al}$$

The stress–strain state effects the austenite transformation ratio, which is manifested as a difference in the stability of the austenite. The influence of the stress–strain state on the stability of the austenite can be extracted by the value of *k* in Equation (3) [24].

(3) $$k\varepsilon =-In\;\left(\frac{f_{\gamma }}{f_{\gamma }{}_{0}}\right)$$

where *f*_γ_, *f*_γ0_, and *k* are the retained austenite content at true strain *ε*, the initial retained austenite content, and the mechanical stability of the retained austenite, respectively. Generally, high retained austenite stability exhibits a low *k* value. The results of combining Figure 3 and Equation (3) to calculate the *k* values of CQ1-ART, CQ2-ART, and CQ3-ART were 2.13, 2.40, and 0.87, respectively. The calculation results show that CQ3-ART exhibited the highest retained austenite stability.

**Figure 5:**
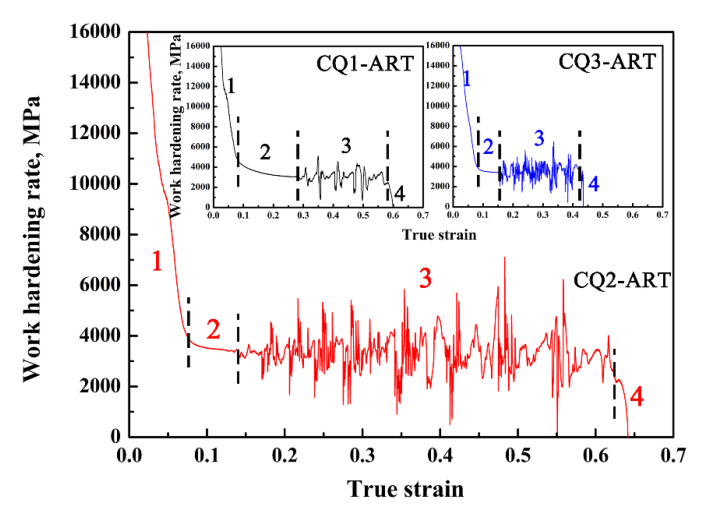
Work hardening rate curves of experimental steels with different numbers of quenching cycles.

In addition, by analyzing the work hardening rate of the experimental steel, we found the reason for the difference in the austenite stability of the experimental steel under different numbers of quenching cycles. Figure 5 shows the work hardening rate curves of the experimental steel; we found that the work hardening rate curves all show four stages of S1, S2, S3, and S4. The decrease in the work hardening rate in the S1 stage mainly related to a large amount of yield deformation by the soft phase ferrite at the initial stage of plastic deformation [25,26]; as the deformation increased, some retained austenite with poor stability first underwent stress-induced martensitic transformation (SIMT) [27], and then the generation of the successive TRIP effect increased material strength, which overcame the softening effect of ferrite, resulting the work hardening rate in the S3 stage to appear as a fluctuating rising stage [28,29]. In the S4 stage, the strengthening effect of the TRIP effect could not offset the failure of a large number of plastic deformation structures, resulting in the work hardening rate decreasing rapidly [30]. For the results of this experiment, the work hardening rate decreased with transitional fluctuations initially in the S4 stage, and then rapidly decreased when the material failed and fractured. It can be seen that there were a few retained austenite discontinuous SIMTs that occurred at the end of the strain. To summarize, the four stages of the work hardening rate had a strong relationship with the stability of the retained austenite, while the CQ3-ART sample was unable to exhibit an extensive TRIP enhancement effect due to the extreme stability of its retained austenite, resulting in a low work hardening rate.

Haidemenopoulos et al. [22] established a model related to austenite stability, which can be used to estimate $M_{S}^{\sigma }$ temperature. It is suggested that the $M_{S}^{\sigma }$ point is mainly a function of chemical composition (chemical driving force of martensitic transformation), yield strength (mechanical driving force of martensitic transformation), grain size (number of potential nucleation points in grains), and stress state (volume change and stress interaction). The chemical driving force for martensite transformation is related to chemical composition and temperature. The chemical driving force can be calculated by Equation (4):

(4) $$\Delta G^{ch}=-7381.6+69447X_{C}+19296X_{Mn}-38776X_{C}X_{Mn}+6.7821T-33.45X_{C}T$$

where Δ*G^ch^* is the chemical driving force; *X_C_* and *X_Mn_* are the mole fractions of *C* and *Mn*, respectively; and *T* is the temperature. The mechanical driving force is a function of the stress state, which can be calculated by Equation (5):

(5) $$\Delta G^{\sigma }=\sigma \left(\frac{\partial \Delta G}{\partial \sigma }\right)=\sigma \left(-0.715-0.3206\left(\frac{\sigma_{h}}{\bar{\sigma }}\right)\right)$$

where Δ*G^σ^* is the mechanical driving force, *σ* is the stress, *σ_h_* is the static horizontal stress, and $\bar{\sigma }$ is the average stress. For uniaxial tension, $(\sigma_{h}/\bar{\sigma })=1/3$. The friction work of interface motion is a function of chemical composition. When the friction work is equal to the critical thermodynamic driving force at the temperature of M_S_, it can be calculated by Equation (6) [31]:

(6) $$W_{f}=1169+8777X_{C}+2246X_{Mn}+19900X_{C}X_{Mn}$$

where *W_f_* is the interfacial friction work (J/mol), and *W_f_* increases with the increase in *C* and *Mn* contents. Stress-assisted martensite nucleation is affected by potential nucleation distribution. Research by Ghosh et al. [32] showed that the defects in the crystal exist in the form of dislocations at grain boundaries and phase interfaces. The decomposition of dislocations produced stacking faults, and some of them provided nucleation sites for martensitic transformation [33]. In unit area, the stacking fault energy *γ_f_*(*n*) needed to produce *n* atomic planes can be calculated by Equation (7) [34]:

(7) $$\gamma_{f}\left(n\right)=n\rho \left[\Delta G^{ch}+E^{str}+W_{f}\right]+2\gamma_{s}$$

where *γ_S_* is the interface energy of martensite nucleation, *ρ* is the atomic density of the close-packed plane, and *E^str^* is the elastic deformation energy in the grain. When *γ_f_*(*n*) ≤0, the critical crystal plane number *n* where the stacking fault occurs is used to represent the potential distribution of nucleation points, which is calculated by Equation (8):

(8) $$n=-\frac{2\cdot \gamma_{s}/\rho }{\Delta G^{ch}+E^{str}+W_{f}}$$

If the number density *Nv* of potential nucleation sites is randomly distributed in the whole crystal, *N*_v_(*n*) can be defined as the potential distribution (*n*) of martensite nucleation sites during martensitic transformation for cooling, and the potential distribution of accumulated structural defects can be calculated by Equation (9):

(9) $$N_{V}=N_{V}^{0}\exp(-\alpha \cdot n)$$

where $N_{V}^{0}$ is the total potential nucleation point, and *α* is a constant. The thermodynamic driving force Δ*G* is introduced to analyze the effect of stress on the potential distribution of nucleation points:

(10) $$\Delta G=\Delta G^{ch}+\Delta G^{\sigma }$$

Combined with Equation (9), the potential distribution probability of nucleation points *N_V_*_(*σ*)_ under stress can be expressed as:

(11) $$N_{V}\left(\sigma \right)=N_{V}^{0}\exp\frac{2\alpha \gamma_{s}/\rho }{\Delta G^{ch}+\Delta G_{max}^{\sigma^{str}{}_{f}}}$$

where $\Delta G_{\max}^{\sigma }$ is the maximum mechanical driving force. The volume fraction of stress-induced transformation from austenite to martensite can be calculated by the amount of density accumulated at the nucleation point and the average austenite grain size:

(12) $$f=1-\exp(-N_{V}\cdot V_{p})$$

where *f* is the volume fraction of austenite transformed into martensite, and *V_p_* is the average grain volume of austenite. When martensite nucleates, the applied stress σ reaches *σ_t_*, combining equations from (7) to (11), the following Equation (13) can be obtained:

(13) $$\sigma_{t}=\frac{1}{\left(\frac{\partial \Delta G}{\partial \sigma }\right)}\cdot \left\{\frac{2\alpha \gamma_{s}/\rho }{\mathrm{In}\left[-\frac{\mathrm{In}\left(1-f\right)}{N_{V}^{0}\cdot V_{p}}\right]}-\Delta G^{ch}-E^{str}-W_{f}\right\}$$

It can be seen from equation (12) that when the applied stress *σ_t_* is equal to the yield strength *σ_y_*, it can be determined. Therefore, substituting Equations (4)–(6) into (12) can be used to obtain the temperature of $M_{S}^{\sigma }$:

(14) $$M_{S}^{\sigma }={\left(6.7891-33.45X_{C}\right)}^{-1}\cdot \left(\begin{array}{l} \frac{2\cdot \alpha \cdot \gamma_{s}/\rho }{\mathrm{In}\left\{-\left[\mathrm{In}\left(1-f\right)\right]/N_{V}^{0}\cdot V_{P}\right\}}+5712.6-78224\cdot X_{C}-\\ 21542\cdot X_{Mn}+18876\cdot X_{C}\cdot X_{Mn}+\sigma_{y}\cdot \left(0.715+0.3026\cdot \frac{\sigma_{h}}{\bar{\sigma }}\right) \end{array}\right)$$

where α = 0.866, *γ_s_* = 0.15 J/m^2^, *ρ* = 3 × 10^−5^ mol/m^2^, *f* = 0.11, $N_{V}^{0}$ = 2 × 10^17^ m^−3^ and E^str^ = 500 J/mol. In summary, the yield strength and grain size of the retained austenite significantly affect the $M_{S}^{\sigma }$ temperature. Low yield strength and fine retained austenite grains result in a low $M_{S}^{\sigma }$ temperature, which in turn exhibits a high retained austenite stability; conversely, a higher $M_{S}^{\sigma }$ temperature causes a low retained austenite stability. Deformation-induced martensite transformation improves the strength and ductility of the experimental steel. At the same time, the improvement in mechanical properties is closely related to austenite stability. Increasingly stable retained austenite is obtained by controlling the $M_{S}^{\sigma }$ point of the experimental steel below the service temperature, a more extensive TRIP effect is produced, and the comprehensive properties of the experimental steel are improved.

**Figure 6:**
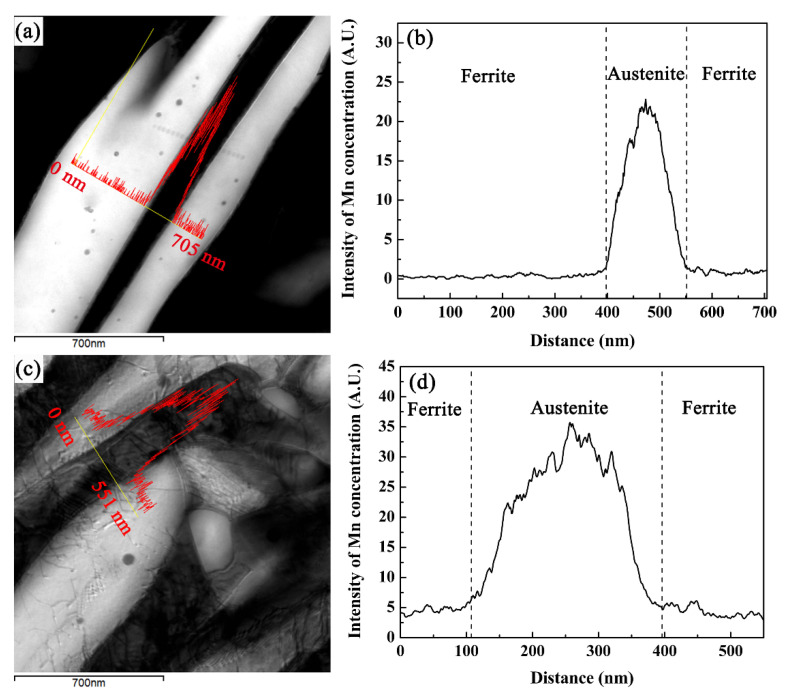
TEM microstructure and corresponding Mn distribution of (**a**,**b**) CQ2-ART and (**c**,**d**) CQ3-ART samples.

Figure 6 shows the TEM microstructure and corresponding Mn distribution of the CQ2-ART and CQ3-ART samples. In order to clarify the influence of chemical elements and grain size on retained austenite stability, TEM-EDS was used for CQ2-ART (average grain size of 0.40 μm) and CQ3-ART (average grain size of 0.42 μm) in the analysis of the Mn content. It can be seen from Figure 6 that the distribution of Mn was uneven in a single retained austenite grain, and the Mn concentration intensity in retained austenite was significantly higher than that in ferrite, which indicated that there is an obvious Mn distribution behavior between ferrite and austenite. In addition, comparing the CQ2-ART and CQ3-ART samples, the former had a finer retained austenite grain size than the latter, while at the same time, the Mn concentration in the latter was significantly higher than the former. Combined with the analysis results in Figure 3 and Figure 5, it is considered that part of the retained austenite in the CQ3-ART sample may have had an extremely high Mn content, showing extremely high stability, leading to difficultly for the retained austenite to undergo martensite transformation during the strain process. Therefore, grain size is not the main factor affecting retained austenite stability in cold-rolled samples after different numbers of quenching cycles, while the Mn content in the austenite is the key factor.

**Figure 7:**
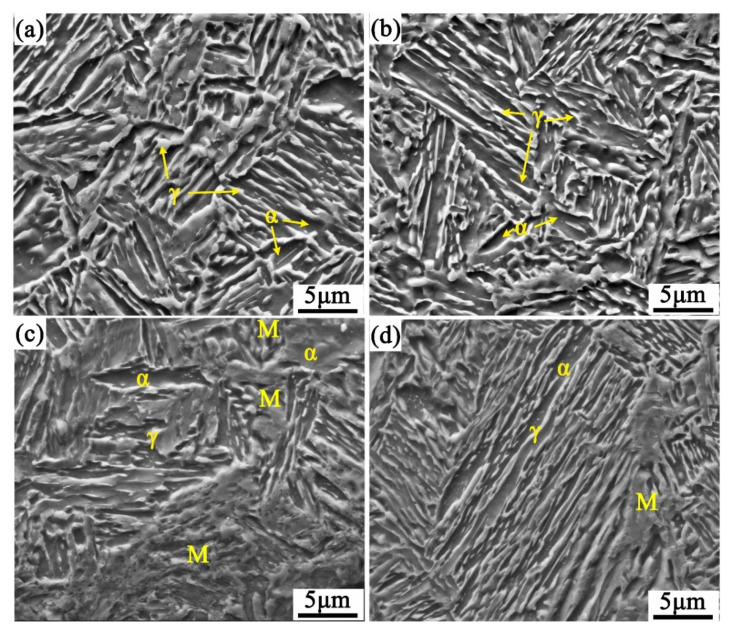
SEM micrographs of CQ2-ART (**a**) and CQ3-ART (**b**) samples before the tensile tests; (**c**,**d**) the corresponding samples after the tensile tests.

Figure 7 shows the SEM microstructure of the CQ2-ART and CQ3-ART samples before and after the tensile tests. It can be seen from Figure 7a,c that the CQ2-ART sample underwent significant strain-induced austenite to martensite transformation during the tensile test; at the same time, the ferrite grain exhibited a concave shape before the tensile test, while part of the ferrite grain had a protruding feeling after the tensile test. It is considered that retained austenite belongs to an FCC crystal lattice, while martensite belongs to a BCC crystal lattice, since retained austenite has a more compact atomic arrangement and higher C dissolving ability than martensite, and the transformation from austenite to martensite leads to the supersaturation of carbon atoms in a martensite lattice; thus, volume expansion occurs when FCC transforms to BCC. The transformation from austenite to martensite results in volume expansion during the tensile process; thus, ferrite around the martensite phase after transformation is subjected to extrusion deformation caused by the increase in martensite, and the resulting deformation produces a sense of protrusion. This deformation process of ferrite offsets the concentration of internal stress, thereby improving the plasticity of the material. However, according to the SEM microstructure of the CQ3-ART sample before and after the tensile test shown in Figure 7b,c there was still some strip-shaped and granular retained austenite in the sample structure after tensile fracture. Compared with the retained austenite after the tensile fracture of CQ2-ART, the retained austenite in CQ3-ART obviously increased. These results further prove that excessively stable retained austenite is not conducive to an extensive TRIP effect, which is consistent with the result of the retained austenite conversion calculated in Figure 3c.

### 3.3. Discontinuous TRIP Effect

**Figure 8:**
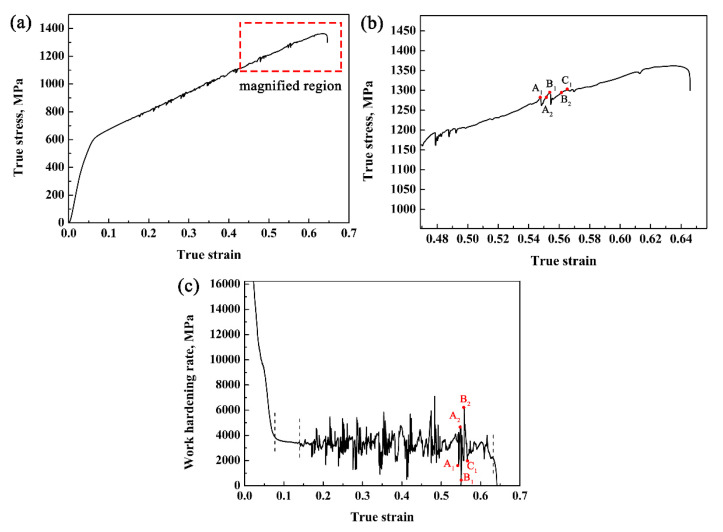
Work hardening behavior and true stress–strain curve: (**a**) true stress-strain curve; (**b**) partial magnification of the true stress-strain curve; (**c**) work hardening rate.

The research of Bhadeshia et al. [35] and Chiang et al. [36] showed that retained austenite stability is affected by its morphology, and the authors considered that the stability of bulk, strip, and granular retained austenite increases in order. It can be seen from Figure 7c that after tensile fracture, the retained austenite grains mostly existed in small strips or granules, while the massive or bulky austenite was transformed into martensite. Simultaneously, it can be seen from Figure 7a that ferrite divided austenite into strips with different thicknesses and lengths, thus having different levels of stability. Figure 8 shows the work hardening rate and true stress-strain curve of the CQ2-ART sample. The true stress-strain curve is shown in Figure 8a, and it can be seen that there are certain large amplitude and sparse sawtooth fluctuations on the curve. Koyama et al. [37] considered that the serrated fluctuation on the curve could be attributed to dynamic strain ageing. Kubin et al. [38] and Qian Kuangwu et al. [39] considered that dynamic ageing is a strengthening phenomenon caused by the interaction between moving solute atoms and dislocations. Figure 8b was obtained by local magnification of the obvious sawtooth fluctuations of the true stress-strain curve in Figure 8a. We found that the fluctuation process was accompanied by a sudden drop and rapid rise in true stress.

Combined with the work hardening rate and true stress-strain curve of the CQ2-ART sample given in Figure 8b,c, the discontinuous TRIP effect is elaborated. The true stress peak value can be divided into two stages from A1 to B1 (Figure 8b), i.e., A1 to A2 and A2 to B1. A1 to A2 belong to the stage of strain-induced transformation from retained austenite to martensite, and the corresponding work hardening rate curve rises rapidly in this stage (Figure 8c). It is considered that when the true stress reached the critical value A1, at which the retained austenite can undergo transformation at the corresponding stability level, the martensite transformation activated to produce the TRIP effect. The enhanced TRIP effect counteracted part of the true stress and produced stress relaxation; at the same time, the stress transferred to the surrounding ferrite and retained austenite, which led to the formation of the surrounding ferrite, and the retained austenite with the same level of stability continued to undergo phase transformation. This increasing external force competed with the stress relaxation caused by the strengthening effect of the TRIP effect, and ultimately led to a temporary sudden drop in true stress. From A2 to B1, it belonged to the stage of continuous increase and accumulation of external stress, which was accompanied by a rapid decrease in the work hardening rate. When the increase and accumulation of external stress reached the critical point B1 for transformation of retained austenite in the next higher stability batch, a new round of strain-induced transformation from retained austenite to martensite occurred, resulting in a TRIP effect. In summary, the key to the discontinuous TRIP effect of the experimental steel lies in the following: Firstly, there must be a certain amount of retained austenite in the experimental steel, which transforms into martensite during the tensile process and causes volume expansion, thus offsetting part of the stress, concentrating and transferring the stress to the surrounding phases, resulting in collaborative deformation accompanied by stress relaxation and transfer. Secondly, the retained austenite in the experimental steel needs to have the stability of different grades of batches, and the TRIP effect can be produced only when the stress value reaches the critical value of the retained austenite transformation of the batch.

Obviously, the Fe-0.25C-3.98Mn-1.22Al-0.20Si-0.19Mo-0.03Nb medium Mn steel designed in this study had a total alloy content of less than 6 wt%. After adopting the cyclic quenching-ART process, it showed excellent comprehensive mechanical properties and austenite with high content and appropriate stability was obtained, which has great industrial application prospects. Contrasting with Ref. [40], most previous studies used more than 5 wt% of manganese to improve the mechanical properties of medium-manganese steel. The 4Mn-Nb-Mo low-alloy experimental steel designed in this study achieves the goal of minimizing the content of total alloying elements while having obvious comprehensive performance advantages.

## 4. Conclusions

In this paper, the stability of austenite and TRIP effect in Fe-0.25C-3.98Mn-1.22Al-0.20Si-0.19Mo-0.03Nb medium Mn steel were studied. The main conclusions of the present work are as follows:

(1) The content of Mn in retained austenite is the main factor affecting its stability. In addition, retained austenite with different grain sizes and Mn contents has different grades of retained austenite stability.
(2) The large fluctuation in the work hardening rate curve in the S3 stage is attributed to the discontinuous TRIP effect. The key to the discontinuous TRIP effect is that a certain amount of retained austenite is required in the experimental steel, and the retained austenite should have the stability of different grades and batches.
(3) The $M_{S}^{\sigma }$ point is affected by the chemical composition, grain size, yield strength, and stress state of the retained austenite. Increasing amounts of stable, retained austenite can be obtained by controlling the temperature of the experimental steel Ms below the service temperature, a more extensive TRIP effect can be produced, and the comprehensive properties of the experimental steel can be improved.

## Figures and Tables

**Table 1:** Chemical Compositions of the Investigated Steel (wt%).

C	Mn	Al	Si	Mo	Nb
0.25	3.98	1.22	0.20	0.19	0.03

## Data Availability

Not applicable.

## References

[1] Dutta A., Park T.M., Nam J.H., Lee S.I., Hwang B., Choi W.S., SandlÖbes S., Ponge D., Han J. (2021). Enhancement of the tensile properties and impact toughness of a medium-Mn steel through the homogeneous microstrain distribution. Mater. Charact..

[2] Yang Y., Mu W., Sun B., Jiang H., Mi Z.L. (2021). New insights to understand the strain-state-dependent austenite stability in a medium Mn steel: An experimental and theoretical investigation. Mater. Sci. Eng. A.

[3] Tian C., Guo H., Hu B., Enomoto M., Shang C.J. (2021). Influence of nano-scale concentration gradient of alloying elements on the ductility in an intercritically annealed and tempered medium Mn steel. Mater. Sci. Eng. A.

[4] Patra A.K., Athreya C.N., Mandal S., Kumar K.C.H. (2021). High strength-high ductility medium Mn steel obtained through CALPHAD based alloy design and thermomechanical processing. Mater. Sci. Eng. A.

[5] Zhao B.G., Wang Y.F., Ding K., Wu G.Z., Wei T., Pan H., Gao Y.L. (2021). Role of Intercritical Annealing in Enhancing the Cross-Tension Property of Resistance Spot-Welded Medium Mn Steel. J. Mater. Eng. Perform..

[6] He B.B., Huang B.M., He S.H., Qi Y., Yen H.W., Huang M.X. (2018). Increasing yield strength of medium Mn steel by engineering multiple strengthening defects. Mater. Sci. Eng. A.

[7] Li X., Song R.B., Zhou N.P., Li J.J. (2018). An ultrahigh strength and enhanced ductility cold-rolled medium-Mn steel treated by intercritical annealing. Scr. Mater..

[8] He B.B., Hu B., Yen H.W., Cheng G.J., Wang Z.K., Luo H.W., Huang M.X. (2017). High dislocation density–induced large ductility in deformed and partitioned steels. Science.

[9] Jirková H., MaŠEk B., Wagner F.X. (2014). Influence of metastable retained austenite on macro and micromechanical properties of steel processed by the Q&P process. J. Alloys Compd..

[10] Liu C.Q., Peng Q.C., Xue Z.L., Wang S.J., Yang C.W. (2018). Microstructure and Mechanical Properties of Hot-Rolled and Cold-Rolled Medium-Mn TRIP Steels. Materials.

[11] Fischer F.D., Reisner G., Werner E., Tanaka K., Cailletaud G., Antretter T. (2000). A new view on transformation induced plasticity (TRIP). Int. J. Plast..

[12] Cherkaoui M., Berveiller M., Lemoine X. (2000). Couplings between plasticity and martensitic phase transformation: Overall behavior of polycrystalline TRIP steels. Int. J. Plast..

[13] Lee H., Jo M.C., Sohn S.S., Zargaran A., Lee S. (2018). Novel medium-Mn (austenite + martensite) duplex hot-rolled steel achieving 1.6 GPa strength with 20% ductility by Mn-segregation-induced TRIP mechanism. Acta Mater..

[14] Cai Z.H., Ding H., Misra R.D.K., Ying Z.Y. (2015). Austenite stability and deformation behavior in a cold-rolled transformation-induced plasticity steel with medium manganese content. Acta Mater..

[15] Li Z.C., Li X.J., Mou Y.J., Misra R.D.K., Ding H., He L.F., Li H.P. (2020). Tuning austenite stability in a medium Mn steel and relationship to structure and mechanical properties. Mater. Sci. Technol..

[16] Liu C.Q., Peng Q.C., Xue Z.L., Yang C.W. (2019). A Novel Cyclic-Quenching-ART for Stabilizing Austenite in Nb–Mo Micro-Alloyed Medium-Mn Steel. Metals.

[17] Srivastava A.K., Bhattacharjee D., Jha G., Gope N. (2007). Microstructural and mechanical characterization of C–Mn–Al–Si cold-rolled TRIP-aided steel. Mater. Sci. Eng. A.

[18] Jha B.K., Avtar R., Dwivedi V.S. (1987). Applicability of modified Crussard-Jaoul analysis on the deformation behaviour of dual-phase steels. J. Mater. Sci. Let..

[19] Samek L., Moor E.D., Penning J., Cooman B.C.D. (2006). Influence of alloying elements on the kinetics of strain-induced martensitic nucleation in low-alloy, multiphase high-strength steels. Metall. Mater. Trans. A.

[20] Wang J., Zwaag S. (2001). Stabilization mechanisms of retained austenite in transformation-induced plasticity steel. Metall. Mater. Trans. A.

[21] Xie Z.J., Liu Z.F., Misra R.D.K., Shang C.J., Han G., Wang X.L. (2019). Retained austenite stabilisation in low carbon high silicon steel during isothermal holding. Mater. Sci. Technol..

[22] Haidemenopoulos G.N., Vasilakos A.N. (1996). Modelling of austenite stability in low-alloy triple-phase steels. Steel Res. Int..

[23] Lee S., Lee S.J., Cooman B.C.D. (2011). Austenite stability of ultrafine-grained transformation-induced plasticity steel with Mn partitioning. Scr. Mater..

[24] Sugimoto K.I., Kobayashi M., Hashimoto S.I. (1992). Ductility and strain-induced transformation in a high-strength transformation-induced plasticity-aided dual-phase steel. Metall. Trans. A.

[25] Lee C.Y., Jeong J., Han J., Lee S.J., Lee Y.K. (2015). Coupled strengthening in a medium manganese lightweight steel with an inhomogeneously grained structure of austenite. Acta Mater..

[26] Godet S., Jacques P.J. (2015). Beneficial influence of an intercritically rolled recovered ferritic matrix on the mechanical properties of TRIP-assisted multiphase steels. Mater. Sci. Eng. A.

[27] Sun B., Vanderesse N., Fazeli F., Scott C., Chen J., Bocher P., Jahazi M., Yue S. (2017). Discontinuous strain-induced martensite transformation related to the Portevin-Le Chatelier effect in a medium manganese steel. Scr. Mater..

[28] Li Z.C., Ding H., Cai Z.H. (2015). Mechanical properties and austenite stability in hot-rolled 0.2 C–1.6/3.2 Al–6Mn–Fe TRIP steel. Mater. Sci. Eng. A.

[29] Li Z.C., Misra D.K.R., Cai H.Z., Li H.X., Ding H. (2016). Mechanical properties and deformation behavior in hot-rolled 0.2C-1.5/3Al-8.5Mn-Fe TRIP steel: The discontinuous TRIP effect. Mater. Sci. Eng. A.

[30] Li Z.C., Ding H., Misra R.D.K., Cai Z.H., Li H.X. (2016). Microstructural evolution and deformation behavior in the Fe-(6, 8.5) Mn-3Al-0.2 C TRIP steels. Mater. Sci. Eng. A.

[31] Hongbing C., Hsu T.Y. (1986). Thermodynamic prediction of MS and driving force for martensitic transformation in Fe-Mn-C alloys. Acta Metall..

[32] Ghosh G., Olson G.B. (1994). Kinetics of FCC→BCC heterogeneous martensitic nucleation—I. The critical driving force for athermal nucleation. Acta Metall. Mater..

[33] Ghosh G., Olson G.B. (1994). Kinetics of FCC→BCC heterogeneous martensitic nucleation—II. Thermal activation. Acta Metall. Mater..

[34] Haidemenopoulos G.N., Katsama A.I., Aravas N. (2006). Stability and constitutive modelling in multiphase TRIP steels. Steel Res. Int..

[35] Bhadeshia H.K.D.H., Edmonds D.V. (1983). Bainite in silicon steels: New composition–property approach Part 2. Metal. Sci..

[36] Chiang J., Lawrence B., Boyd J.D., Pilkey A.K. (2011). Effect of microstructure on retained austenite stability and work hardening of TRIP steels. Mater. Sci. Eng. A.

[37] Koyama M., Sawaguchi T., Lee T., SooLee C., Tsuzaki K. (2011). Work hardening associated with ε-martensitic transformation, deformation twinning and dynamic strain aging in Fe–17Mn–0.6C and Fe–17Mn–0.8C TWIP steels. Mater. Sci. Eng. A.

[38] Kubin L.P., Estrin Y. (1990). Evolution of dislocation densities and the critical conditions for the Portevin-Le Chatelier effect. Acta Metall. Mater..

[39] Qian K.W., Li X.Q., Xiao L.G. (2001). Dynamic strain aging in metals and alloys. J. Fuzhou Univ..

[40] Hu B., Luo H., Yang F., Dong H. (2017). Recent progress in medium-Mn steels made with new designing strategies, a review. J. Mater. Sci. Technol..

